# The effects on thermal lesion shape and size from bubble clouds produced by acoustic droplet vaporization

**DOI:** 10.1186/s12938-018-0596-z

**Published:** 2018-10-29

**Authors:** Ying Xin, Aili Zhang, Lisa X. Xu, J. Brian Fowlkes

**Affiliations:** 1 School of Biomedical Engineering, 400 Med-X Research Institute, Shanghai Jiao Tong University, 1954 Huashan Rd, Shanghai, China; 2 Department of Radiology, University of Michigan Health System, 3226C Medical Sciences Building I, 1301 Catherine Street, Ann Arbor, MI USA

**Keywords:** Acoustic droplet vaporization (ADV), Bubble cloud, Thermal lesions, HIFU ablation

## Abstract

**Background:**

Bubbles formed by acoustic droplet vaporization (ADV) have proven to be an effective method for significant enlargement of the thermal lesions produced by high intensity focused ultrasound (HIFU). We investigated the influences of bubble cloud shape and droplet concentration on HIFU thermal lesions, as these relate to the ADV technique.

**Methods:**

Unlike previous studies where the droplets were simultaneously vaporized with the HIFU exposure for thermal lesion formation, droplets were vaporized by pulse wave (PW) ultrasound prior to continuous wave (CW) ultrasound heating in this experimental study. Under different experimental conditions, we recorded and quantified by the image processing methods the morphology and size of the bubble clouds created and the corresponding thermal lesions formed.

**Results:**

The results demonstrated that different ADV droplet concentrations produced a variety of thermal lesion shapes and sizes. The lesion volume could be increased using PW ultrasound followed by CW exposure, especially for higher droplet concentrations, e.g. 3.41 × 10^6^/mL yielded a tenfold increase over that seen using CW alone.

**Conclusion:**

These findings could lead to optimization of HIFU therapy by selecting a bubble forming strategy and droplet concentrations, especially using lower ultrasound powers which is desirable in clinical applications.

**Electronic supplementary material:**

The online version of this article (10.1186/s12938-018-0596-z) contains supplementary material, which is available to authorized users.

## Introduction

High intensity focused ultrasound (HIFU) treatment is gaining popularity due to its noninvasiveness. By focusing ultrasound energy in a small area inside the body, the temperature can be elevated to over 56 °C in seconds and cause irreversible necrosis and damage of the tumor tissue [1]. However, due to the need to protect normal tissue and the small lesion size from one single application of HIFU, the process is time consuming, especially for large tumors which may require a treatment time of several hours [1, 2].

To shorten the treatment time, researchers have tried to use bubbles to help increase local heating [3–9]. Microbubbles can be introduced into tissue by injection of ultrasound contrast agents (UCA) containing bubbles, using high rarefactional pressure to trigger cavitation for generation of bubble nuclei or by using acoustic droplet vaporization (ADV) where liquid droplets are injected and then vaporized to bubbles in the HIFU focal region [10]. Among these techniques, use of UCA and ADV droplets requires lower acoustic pressure. While the bubbles are delivered to the whole body of the patient when using UCA, the ADV droplets technique ensures that the bubbles only exist in the focal region and therefore eliminates the possible shift of the HIFU focus and other effects due to the UCA bubbles being throughout the ultrasound propagation path [6].

Phantom and animal experiments have confirmed that ADV-assisted HIFU can achieve a 3–15 times larger lesion than HIFU alone [11, 12], so the number of HIFU exposures required to treat the whole tumor can be significantly reduced. The bubbles formed in the HIFU focal region by this technique help increase heat deposition based on three mechanisms: (1) bubble oscillations driven by ultrasound, and corresponding viscous heating; (2) the high frequency mechanical waves generated by the bubble oscillation; (3) the trapping of the ultrasound in the bubble network and the correspondingly lengthened propagation path and increased absorption of the tissue. Meanwhile, as bubbles are so efficient at converting energy, the ultrasound energy attenuates much faster along the propagation path in the presence of bubbles, leading to much lower ultrasound energy at distal locations [6, 13, 14]. From the mechanisms of bubbles increasing heat deposition, it could be easily speculated that the size and morphology of the thermal lesions are dependent on both the nature of the bubble region (morphology of the region, the bubble size and concentration, etc.) and the ultrasonic field.

In addition to triggering the droplets in the HIFU focus to help increase thermal absorption, ADV can create a bubble shield against ultrasound wave propagation to protect normal tissue [13], or to further confine the ultrasound wave in the targeted treatment region [15]. The high impedance difference of the bubble region leads to more reflection and scattering over the bubble cloud surface and the redistribution of the ultrasound energy. The acoustic impedance (density × sound speed) of the bubble region is dependent on bubble size distribution, bubble concentration and the ultrasound frequency [16].

From these previous studies, we can see that the bubble region dimension and the density and size of the bubbles inside are critical to the shape of the subsequent thermal lesions formed by HIFU. As the ADV droplets can provide a way to create desired shapes of bubble clouds, understanding how the local bubble region characteristics may influence the thermal lesions by HIFU would be essential for planning a much more efficient ADV enhanced HIFU treatment strategy. Meanwhile, it also provides a way to treat the tumor according to the actual tumor shape, while protecting the critical, normal tissue structures outside the tumor.

Thus, in this study, different shapes of bubble clouds were formed by the ADV technique with pulse wave (PW) ultrasound from the same transducer prior to the continuous wave (CW) HIFU thermal treatment. In this paper, we analyze and discuss the influences of droplet concentration and acoustic pressure amplitude on the shape and volume of the bubble clouds and associated thermal lesions.

## Materials and methods

### Experimental setup

**Fig. 1:**
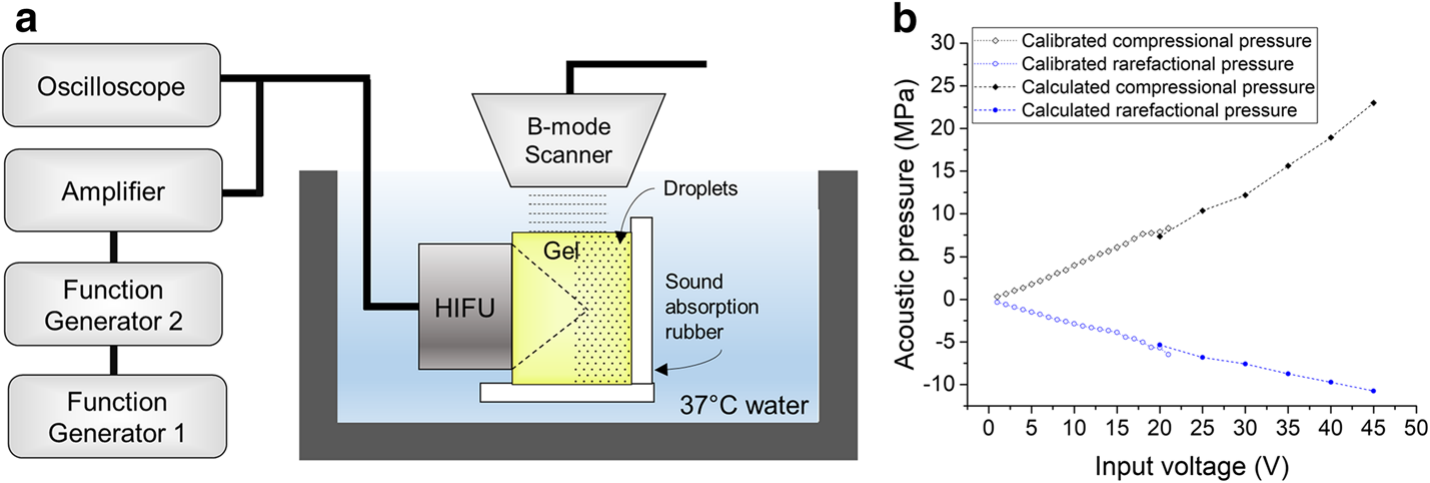
**a** Experimental setup. The HIFU transducer was driven by the RF amplifier. The input voltage to the transducer was monitored by an oscilloscope. Two function generators were used to send a certain number of pulses or a certain duration of continuous wave RF signals to the amplifier. **b** The output pressure of the transducer as function of input voltage. For applied voltages of 1–21 V, the transducer was calibrated by a hydrophone. For higher input voltages, the pressures were calculated by a modified KZK equation [17]

The experimental setup is illustrated in Fig. 1a. The HIFU transducer was driven by the RF amplifier (AG1006, T&C Power Conversion, Rochester, NY). The input voltage to the transducer was monitored by an oscilloscope (MSO-X 2022A, Agilent Technologies, Inc., Santa Clara, CA). Two function generators (33210A, 33220A, Agilent Technologies, Inc., Santa Clara, CA) were used to send RF signals to the amplifier. One function generator gated the other to assure that signals of a certain number of pulses or a certain duration of continuous wave RF were input to the amplifier.

The HIFU transducer used in the study was a single element spherically focused transducer (H-108, Sonic Concepts, Inc., Bothell, USA), with a center frequency of 2.7 MHz. The aperture and focal length of the transducer were 60 mm and 50 mm, respectively. The transducer was calibrated for applied voltages of 1–21 V using a fiber optic hydrophone (FOH/1, Precision Acoustics Ltd., UK). For the higher input voltages, the forward electrical power was measured by an internal circuit in the power amplifier and the acoustic power from the transducer was calculated based on the transducer specification of its efficiency. The acoustic power was used to calculate the focal pressure using a modified KZK equation for low F-number transducers [17]. The output pressure of the transducer is a function of input voltage and its peak positive and negative values are shown in Fig. 1b. For the 150 V pulse wave, the electric power was extrapolated from the measured electric power at lower voltages (20–60 V). The corresponding focal pressure was then calculated from the modified KZK equation with this input power. As the transducer doesn’t fully ring up at the three cycles pulses (the PW mode), the calculated pressure was scaled by the same parameter obtained from the calibration for lower input voltages (0.87 for peak positive and 0.85 for peak negative). Due to the nonlinear nature of the ultrasonic waveform, the input voltage is used in the following text. The corresponding acoustic pressures in gel phantom were also calculated, and listed in Table 1 with comparison to those in water.

**Table 1:** Estimated focal pressures of H-108 in water and in gel using a modified KZK equation [17]

Input voltage V	In water		In gel	
	Compressional pressure MPa	Rarefactional pressure MPa	Compressional pressure MPa	Rarefactional pressure MPa
20	7.3	− 5.3	5.0	− 3.7
25	10.4	− 6.8	7.0	− 4.7
30	12.2	− 7.6	8.3	− 5.3
35	15.6	− 8.7	10.6	− 6.1
40	18.9	− 9.7	12.8	− 6.8
45	23.0	− 10.7	15.5	− 7.5
150	136.9	− 15.9	105.8	− 13.8

The water density, the sound speed and sound attenuation coefficient in water used for calculations were 993 kg/m^3^, 1523.7 m/s [18] and 0.04 Np/m [19], respectively. The phantom density, sound speed and sound attenuation coefficient in phantom used were 995 kg/m^3^ [20], 1543 m/s [20] and 6.8 Np/m [20], respectively

The transducer and the phantom samples were immersed in 37 °C water. The focus of the transducer was positioned at a depth of 40 mm in the phantom. A B-mode scanner (MyLab 90, Esaote, Italy) was aligned with the focal axis of the HIFU transducer to monitor and record the bubble cloud created. The B-mode scanner was set to operate at the lowest output power to avoid any effects during the experiments. The images of the phantom were recorded both before and after PW and/or CW ultrasound exposure.

### Preparation of perfluoropentane droplets and tissue-mimicking phantom

Lipid coated perfluoropentane droplets were prepared according to a previously published method [10]. A 750 μL volume of lipid blend was made by dissolving 1.9 mg DPPC (1,2-dipalmitoyl-*sn*-glycero-3-phosphocholine, 850355P, Avanti Polar Lipids, Inc., Alabaster, AL) and 0.08 mg DPPA (1,2-dipalmitoyl-*sn*-Glycero-3-Phosphate, 830855P, Avanti Polar Lipids, Inc., Alabaster, AL) in propylene glycol and 8:1 (v/v) saline-glycerol (Glycerol, Shanghai Chemical Reagent Co,. Ltd., China). Then 250 μL perfluoro-*n*-pentane (09-6182, Strem Chemicals, Inc., Newburyport, MA) was added to the lipid blend. The resulting mixture was sonicated for 30 s to produce the droplet emulsion. The droplet size distribution of the emulsion was measured by a Coulter counter (Multisizer 3, Beckman Coulter, Inc., Fullerton, CA) and can be found in Additional file 1. The droplet concentration in the emulsion was 3.41 × 10^10^/mL, and the resulting mean diameter of the droplets was 0.89 µm.

Tissue-mimicking phantoms that can visualize thermal lesions were made by the same procedure as in previously published literature [20]. The phantom consists of 31.4% (v/v) water, 35% (v/v) egg white, 33% acrylamide solution (30% w/v, 19:1 acrylamide/bis-acrylamide, B161054, Decent Biotech (Shanghai) Co., Ltd., China), 0.5% of 10% (w/v) ammonium persulfate (Shanghai Maibio Co., Ltd, China) and 0.1% TEMED (*N*,*N*,*N*′,*N*′-tetramethylethylenediamine, 15524010, Thermo Fisher Scientific Inc., Waltham, MA). The gel solution was degassed to remove potential gas nuclei. Different volumes of droplet emulsion were added to the gel solution to achieve different droplet concentrations inside the phantom. Each phantom had a size of 120 mm × 70 mm × 50 mm. The transducer was focused at a depth of 40 mm (along the 50 mm dimension) from the front surface of the phantom for each HIFU exposure. For all phantoms containing droplets, to eliminate any possible scattering of the droplets along the ultrasound wave propagation path, the phantom was composed of two halves, one proximal half was free of droplets, while the distal half of the phantom had the desired droplet concentration (as illustrated in Fig. 1a). The HIFU transducer was focused at the distal half, with each exposure placed at least 10 mm apart to avoid interference from each other.

### Treatment strategies

Two treatment strategies were used in the experiments to investigate various characteristics of the bubble clouds and thermal lesions created in phantoms with different droplet concentrations. In the first strategy, the phantom was only exposed to 10 s of continuous wave ultrasound (CW alone). In the second strategy, the phantom was first exposed to a short pulse wave ultrasound to vaporize the ADV droplets in the phantom, then it was exposed to the 10 s of continuous wave to create thermal lesion (PW combined CW). Six different applied voltages were used for the CW ultrasound, 20 V, 25 V, 30 V, 35 V, 40 V and 45 V. The pulse wave used to vaporize droplets was 200 pulses with a pulse repetition frequency of 1 kHz, each pulse consisting of three cycles at 150 V. Five droplet concentrations were investigated in the experiment, 1.07 × 10^5^/mL, 3.41 × 10^5^/mL, 1.07 × 10^6^/mL, 3.41 × 10^6^/mL and 1.07 × 10^7^/mL. Control experiments were conducted in phantoms free of droplets, which were exposed to the CW ultrasound at 30 V for 10 s and to 40 V for 60 s.

### Measurement and data analysis

**Fig. 2:**
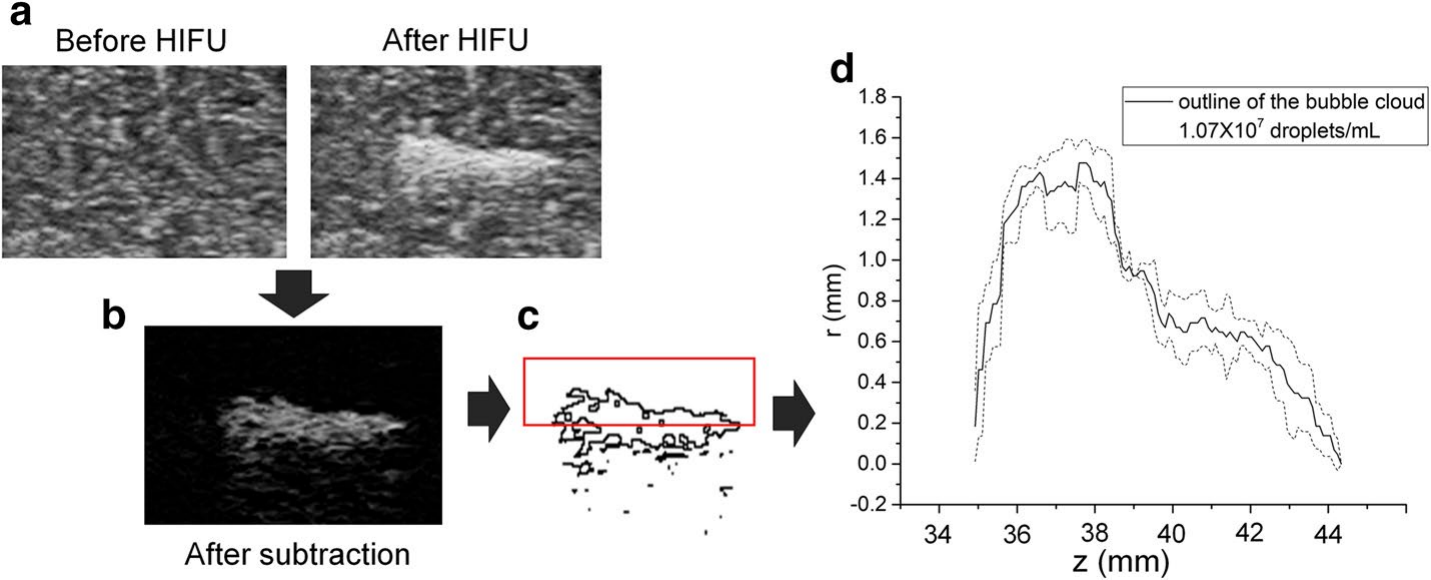
**a** B-mode images of the phantom before and after HIFU treatment with the associated bubble cloud appearing in the latter. **b** The subtraction of the two images in (**a**). **c** The outline of the bubble clouds determined using methods described in the text. The red square marks the upper half of the bubble cloud. **d** The averaged outline over repeated experiments was obtained (solid line). The dashed lines represent the averaged outline ± one standard deviation of the outline (n ≥ 4)

All B-mode images were converted to 8-bit images. Images of the same phantom before and after a given ultrasound exposure were subtracted. Then the threshold method of Isodata [21] was used to determine the outlines of the bubble clouds. The procedure divides the image into object and background by using an initial threshold, then calculates the averages of the pixels at or below the threshold and as well as those pixels above the threshold. The average of these two values are calculated, the threshold increased and the process repeated until the threshold is larger than the composite average. In the cases of high droplet concentration, the B-mode images following ADV attenuate significantly along the direction of the B-mode ultrasound propagation. Therefore, the lower part of the bubble cloud cannot be clearly seen and the upper outlines of the bubble clouds were quantified. This was acceptable as the bubble clouds are expected to be symmetric around the propagation axis of the transducer. The method is illustrated in Fig. 2. Then the outlines of the bubble clouds in the repeated experiments were aligned using the matching method of normalized correlation coefficient [22], which is widely used in pattern matching and object recognition. The bubble cloud outlines obtained with the same experimental conditions were averaged, and the standard deviations were obtained accordingly.

The volume of the bubble cloud was calculated by the equation

1 $$V = \int_{0}^{L} {\pi r\left( z \right)^{2} dz}$$

where *L* is the total length of the bubble cloud, and *r(z)* is the outline curve of the bubble cloud.

After the ultrasound exposure and B-mode imaging, the phantom was taken out of the water and dissected to a thickness such that the thermal lesions could be visually observed. To clearly distinguish the thermal lesion from the bubble cloud, the phantom was placed in a container and pressurized to 3 MPa for 1 min. The bubbles in the phantom were thus compressed, leaving thermal lesions clearly seen. The thermal lesions were photographed and the same image processing method for quantifying the shape of the bubble clouds was used to obtain the outlines of the thermal lesions and calculate their volumes. Student’s t-test was used to determine whether there were significant differences between experimental groups.

## Results and discussion

### Effect of pulse wave (PW) and continuous wave (CW) ultrasound on ADV cloud production

During clinical HIFU treatment, to generate enough heating effect, continuous wave (CW) is usually used. In these cases, the droplets could also be vaporized. Then the vaporized bubbles would oscillate and undergo some of the following processes: (1) some collapse violently; (2) some expand to larger bubbles due to rectified diffusion, then may break into several smaller bubbles; (3) some would merge and become larger bubbles. Thus, using continuous wave ultrasound may result in distribution of bubble sizes in the bubble cloud.

**Fig. 3:**
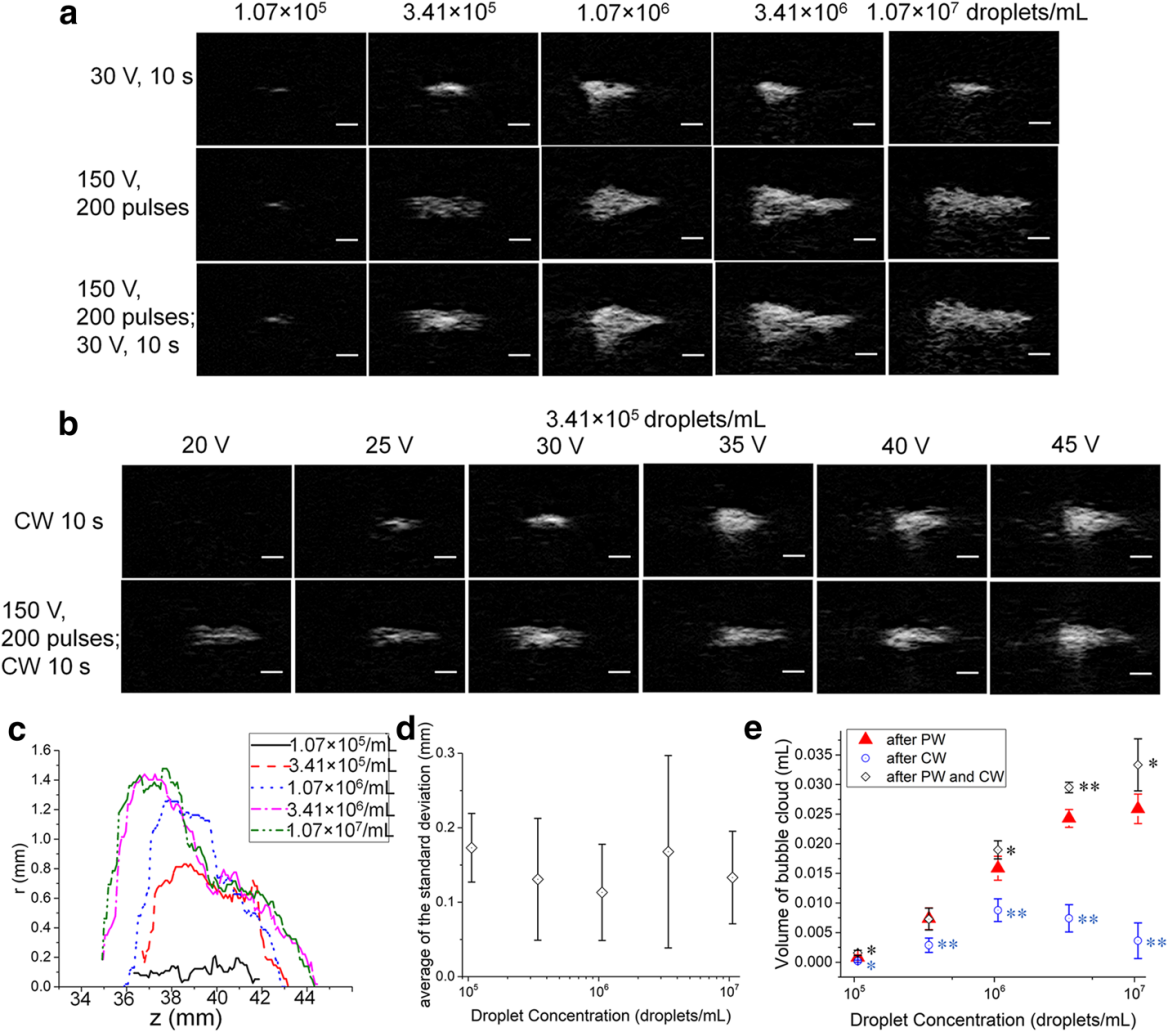
**a** The bubble clouds created after 10 s exposure to continuous wave (CW) at 30 V (row 1), after exposure to 200 pulses (3 cycles each) of pulse wave (PW) at 150 V (row 2), exposure to 200 pulses of pulse wave at 150 V followed by 10 s of continuous wave at 30 V in the phantoms (row 3). The different droplet concentrations used were 1.07 × 10^5^/mL, 3.41 × 10^5^/mL, 1.07 × 10^6^/mL, 3.41 × 10^6^/mL, and 1.07 × 10^7^/mL, respectively. **b** The bubble clouds created after 10 s exposure to continuous wave (row 1), exposure to 200 pulses of pulse wave at 150 V followed by 10 s of continuous wave (row 2). The input voltage of the continuous wave were 20 V, 25 V, 30 V, 35 V, 40 V, and 45 V, respectively. The droplet concentration in the phantom was 3.41 × 10^5^/mL. The scale bar represents 2 mm. **c** The averaged outline of the bubble clouds created after the exposure to 200 pulses of pulsed wave at 150 V in phantom with droplet concentration of 1.07 × 10^5^/mL, 3.41 × 10^5^/mL, 1.07 × 10^6^/mL, 3.41 × 10^6^/mL, and 1.07 × 10^7^/mL, respectively. **d** The averaged standard deviation along bubble cloud outline in phantoms with different droplet concentrations after the exposure to 200 pulses of pulse wave at 150 V. **e** The volume of bubble cloud created in cases of different droplet concentrations after PW and after CW exposure. Colored * and ** represents *p* ≤ 0.05 and *p* < 0.01 for significance of difference between the group of this color and the group of the PW treatment

The bubble clouds created with CW ultrasound alone in phantoms with various droplet concentrations are shown in Fig. 3a (row 1). It can be seen that after the phantoms were exposed to 10 s of CW ultrasound with 30 V input voltage, the size of the bubble clouds initially increased slowly with increasing droplet concentration. The peak volume was reached at the concentration of 1.07 × 10^6^/mL (Fig. 3e) then decreased with further increases in concentration.

In order to study the characteristics of ADV bubble clouds formed by PW ultrasound, the droplets were triggered by the HIFU transducer. The phantoms with different droplet concentrations were exposed to 200-pulse PW with an input voltage of 150 V. Example B-mode images obtained are shown in Fig. 3a (row 2). It can be seen that when the droplet concentration was as low as 1.07 × 10^5^/mL, few droplets were vaporized by the exposure, shown as the several echogenic dots in the B-mode image. In the case of 3.41 × 10^5^/mL, more droplets were vaporized in the focal area resulting in a larger elliptically-shaped bubble cloud. In the case of 1.07 × 10^6^/mL, the bubble clouds had a teardrop shape, with a wider proximal area moving toward to the transducer. In the cases of higher droplet concentration, the bubble clouds had a more triangular shape, the ‘head’ of the bubble cloud is wider and flatter along radial direction, and was closer to the transducer. (At the higher bubble concentrations, the appearance of this cloud in the ultrasound image is likely affected by the attenuation.) The averaged outlines of the bubble clouds taken from the proximal side with respect to the ultrasound imaging are shown in Fig. 3c where the progression of the proximal side of the cloud can be seen as increasingly wider and moving toward the transducer. The average of the standard deviation along the length of the bubble cloud outline was calculated (Fig. 3d) and there was no statistical difference across the droplet concentrations tested, i.e. the width of the transition boundary was similar in all cases. Since the bubble cloud has a larger size in higher droplet concentration cases, the corresponding relative error is lower and therefore better reproducibility is achieved in generating the bubble cloud shape. This could be because at low concentrations, with a limited number of droplets in the focal zone, the bubble clouds created depend much more on the random spatial distribution of the droplets, which varied from case to case. The evolution and variety of bubble cloud shapes for different droplet concentrations might be explained as follows. When the first ultrasound pulse arrives, the acoustic field established in the phantoms is almost the same since the droplets have little influence on the propagation of the ultrasound. But after the first pulse, different numbers of bubbles are created in the same focal area depending on the droplet concentration. A higher bubble density (void fraction) leads to a larger impedance difference, which means more acoustic energy is scattered by the surface of the bubble cloud, so the pressure in front of the bubble cloud is higher in the higher concentration case, leading to a larger volume of bubble cloud after subsequent ultrasound pulses. This implies that less PW power would be required to achieve a same volume of bubble cloud in the presence of a higher droplet concentration, a potentially safer means to the same end.

Compared with the bubble clouds created with the CW only (Fig. 3e), using the PW with an input voltage of 150 V for 200 pulses resulted in a much larger bubble cloud (*p *< 0.01 for droplet concentrations ≥ 3.41 × 10^5^/mL, *p *< 0.05 for all cases). It can also be seen in Fig. 3a (rows 1 and 2), that the echogenicity created using PW are not as great as in the CW case for phantoms with the same droplet concentration. Significant differences were found in the mean gray value of the bubble cloud created after CW and after PW for droplet concentrations from 3.41 × 10^5^/mL to 3.41 × 10^6^/mL (*p* < 0.05). (Comparison of the results for the lowest and highest concentrations may have been affected by the relatively small size of the bubble clouds for the CW cases.) This difference in echogenicity indicates a likely higher impedance difference in those CW cases. Since the initial droplet concentration is the same, this could imply a larger void fraction has been generated. For the CW case, the temperature of phantom increases, so the thermal expansion of the bubbles may lead to a lower impedance. In addition, during the prolonged bubble oscillation, there may be a net inflow of gas into the bubbles from the surrounding medium due to rectified diffusion [23, 24]. And this process could also help bubbles grow larger. In both cases, the bubbles could break up into smaller ones but the overall void fraction would still increase.

Similar effects were observed when PW is combined with the CW as shown in third row of Fig. 3a. These are the same phantoms shown in Fig. 3a (row 2) but exposed to CW immediately after the PW exposure without moving the phantom. After exposure to PW and CW, the pixel value inside the bubble clouds were significantly higher than those exposed to PW alone, in all cases *p* < 0.05. The estimated volume of the bubble cloud is also significantly larger in these cases than in PW only cases when droplet concentrations were 1.07 × 10^5^/mL, 1.07 × 10^6^/mL, 3.41 × 10^6^/mL and 1.07 × 10^7^/mL (*p* ≤ 0.05), which means more bubbles were formed outside of the previous bubble cloud during the CW exposure in these cases. When the droplet concentration was 3.41 × 10^5^/mL, as can be seen from Fig. 3a, the high echogenic regions after CW were all located inside the bubble cloud created by PW only. This causes the insignificant difference between the quantified bubble cloud volume formed by PW only and the combined PW and CW treatment. While for higher droplet concentrations, the bubble density formed from PW only was so high that the acoustic waves from the subsequent CW exposure were scattered outside the preformed bubble cloud and vaporized local droplets and thus resulted in a greatly increased bubble cloud volume. When the concentration of the droplets is as low as 1.07 × 10^5^/mL, the volume of the bubble clouds quantified are very small and there is significant difference among the groups. The quantified volume of bubble cloud formed by PW is larger than that formed by the CW only, but is smaller than the combined exposure. This is different from the case for the droplet concentration of 3.41 × 10^5^/mL because the sparsely distributed bubbles formed by PW are not totally observed by the B-mode image, but are visible after being expanded by the subsequent CW exposure.

The influences of varying the CW voltage are shown in Fig. 3b. The droplet concentration is 3.41 × 10^5^/mL and the phantoms are exposed to CW alone (row 1) and to PW combined with CW (row 2). The input voltages of CW exposures were 20 V, 25 V, 30 V, 35 V, 40 V, and 45 V, respectively. It can be seen that when the input voltage was 20 V, no echogenic area was observed in the B-mode image for the CW alone case, indicating that no droplets were apparently vaporized and the threshold was not achieved even in the focal area. In the cases of 25 V and 30 V, ellipsoidal bubble clouds were created. From 35 to 45 V, the bubble cloud became larger with increasing input voltage, with the ‘head’ of the bubble cloud moving toward the transducer, resulting in a teardrop shaped bubble cloud.

Comparing the bubble clouds created by PW combined with CW to CW alone, these two treatment strategies were quite different when the CW voltage was low. The volume of the bubble clouds created by PW combined with CW was larger than that from CW alone (*p* < 0.05) when the CW input voltage ≤ 30 V. With increasing CW voltage (≥ 35 V), no significant difference was found in the volume of the bubble cloud (*p* > 0.05). No significant difference was found in the mean gray value of the bubble cloud (*p* > 0.05), indicating that applying the PW before the CW has little influence in these cases.

For the cases studied here, it can be seen that bubble clouds of different shapes, sizes and densities can be formed depending on a range of ultrasound parameters and droplet concentrations. The vaporization of droplets is dependent on the pressure amplitude, but the higher pressure amplitude in the short pulse used for PW here should still generate little heating while producing a much larger bubble cloud. Different shapes and sizes over various droplet concentrations were also seen, likely due to the range of impedance differences produced. We have previously shown how the changing impedance in the medium, and the associated scattering, due to the evolving bubble cloud can produce uniquely shaped clouds after a number of pulses are applied [25]. The void fraction of bubbles achieved in CW is also pressure dependent where the additional effects of temperature increases and rectified diffusion may also play a role. It is the dynamic interaction of the acoustic pressure field and the ADV bubbles that lead to a great variety of bubble cloud characteristics. This will definitely affect the lesion shape formed during the HIFU treatment; thus the influence of bubble clouds on the final thermal lesion is examined next.

### Observations on the thermal lesions formation

**Fig. 4:**
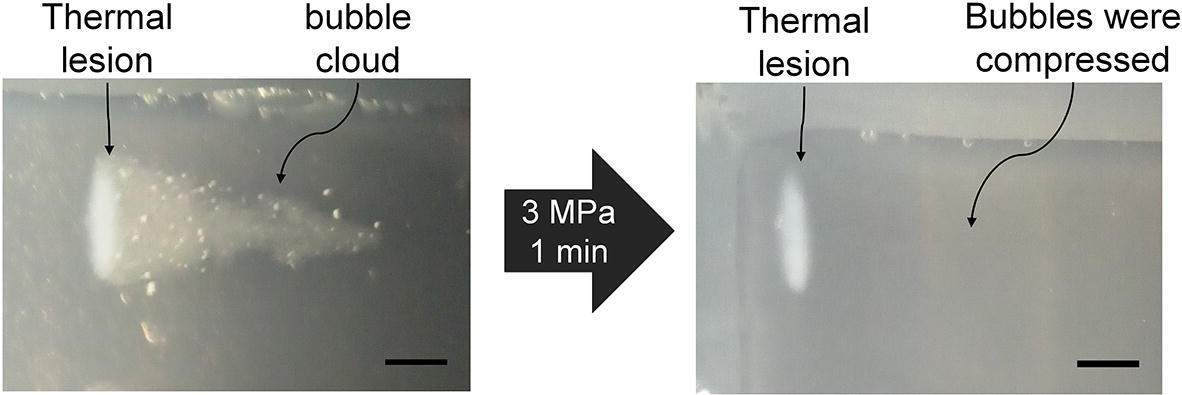
After the HIFU treatment, each phantom was placed in a chamber and pressurized to 3 MPa for 1 min to compress the bubbles, leaving thermal lesion clearly seen

**Fig. 5:**
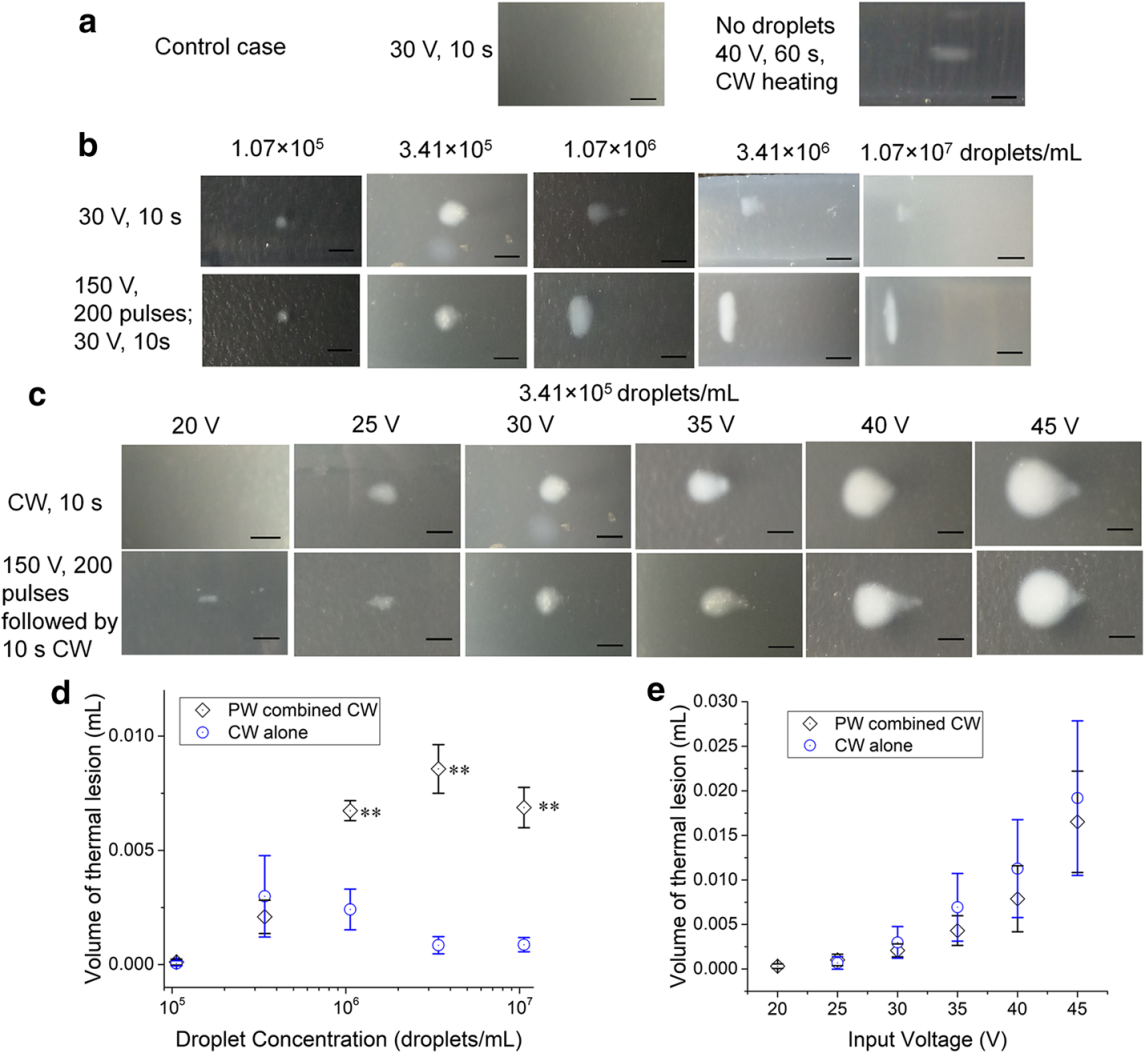
**a** The thermal lesion created in the control case (phantoms without droplets). **b** The thermal lesion created after (row 1) 10 s exposure to continuous wave (CW) at 30 V, (row 2) exposure to 200 pulses of pulse wave at 150 V followed by 10 s of continuous wave at 30 V in the phantoms with different droplet concentrations, which were 1.07 × 10^5^/mL, 3.41 × 10^5^/mL, 1.07 × 10^6^/mL, 3.41 × 10^6^/mL, and 1.07 × 10^7^/mL, respectively. **c** The thermal lesion created after (row 1) 10 s exposure to continuous wave, (row 2) exposure to 200 pulses of pulse wave at 150 V followed by 10 s of continuous wave. The input voltage of the continuous wave were 20 V, 25 V, 30 V, 35 V, 40 V, and 45 V, respectively. The droplet concentration in the phantom was 3.41 × 10^5^/mL. **d** Volume of the thermal lesions as function of droplet concentration (n ≥ 4). **e** Volume of the thermal lesions in phantom with droplet concentration of 3.41 × 10^5^/mL as function of CW input voltage (n ≥ 4). The scale bar represents 2 mm. ** Represents *p* < 0.01 between PW combined with CW and CW alone

The thermal lesions created in the above experiments were also studied to investigate how the different bubble clouds changed the formation of these lesions. Due to the coexistence of the bubbles and the thermal lesion inside the phantom after the HIFU exposures, it is hard to distinguish the lesion from the bubble cloud from direct visual observation. Therefore, all phantoms were subsequently placed in a chamber and a pressure of 3 MPa was applied for 1 min. As shown in Fig. 4, by this technique, the bubbles in the phantom are compressed, leaving thermal lesions clearly visible. The thermal lesions formed in the phantoms were then obtained using this technique and quantified with results shown in Fig. 5.

For comparison purposes, experiments were also conducted in a phantom without any droplets (Fig. 5a). It was found that only after increasing the input voltage to 40 V with a duration of 60 s CW could a small ellipsoidal thermal lesion be observed. This shape is a typical thermal lesion created by HIFU in the absence of bubbles [1]. No echogenic regions were observed in B-mode images of the phantoms, indicating that no cavitation phenomena occurred under these conditions at least at the level of imageable bubble production.

For the thermal lesions formed after 10 s exposure to CW in phantoms with different droplet concentrations (Fig. 5b, row 1), the lesions created had a spherical shape with a small distal extension, and a range of the sizes. The lesion volume reached a peak at the concentration of 3.41 × 10^5^/mL (see Fig. 5d), and then decreased with the increasing concentration. This trend is similar to the bubble cloud volume variation seen in Fig. 3e.

When the phantoms were exposed to the PW before the CW, the shape and position of the thermal lesions changed significantly depending on the droplet concentration, as seen in Fig. 5b (row 2). At the two lowest droplet concentrations, the thermal lesions formed are quite similar to those formed in the CW only group (row 1). And the shape is also similar. But with increasing droplet concentration, the thermal lesions became larger, and the lesions changed from a teardrop to the vertically-oriented ellipsoidal shape in the proximal portion of the focal area. As the droplet concentration reached 3.41 × 10^6^/mL, the thermal lesion size started to decrease and moved closer to the transducer, and the shape got thinner in the axial dimension. With a droplet concentration of 1.07 × 10^7^/mL, the thermal lesions were even thinner, only present in the layer close to the proximal surface of the bubble cloud. This is due to the high attenuation of the high bubble concentration. Thus the acoustic pressure decreases dramatically along the propagation path and only in the first layers of the bubble cloud region is the acoustic energy absorbed and transformed to thermal energy to create a thermal lesion. In addition, the high concentration of bubbles also leads to a lower thermal conductivity, which helps shape the thermal lesion at the proximal area of the bubble cloud and generate these distinct geometries for the thermal lesions.

According to Fig. 5d, the volume of the thermal lesion is significantly increased using the PW combined CW treatment for the high concentration cases (*p *< 0.01 for droplet concentration ≥ 1.07 × 10^6^/mL). With a droplet concentration of 3.41 × 10^6^/mL, the volume created by PW combined CW treatment is more than 10 times the volume created by CW alone.

The thermal lesions formed using different CW input voltages are shown in Fig. 5c. The droplet concentration in the phantom is 3.41 × 10^5^/mL. The duration of continuous wave exposure is 10 s. And the CW input voltages were 20 V, 25 V, 30 V, 35 V, 40 V, and 45 V, respectively. It can be seen in Fig. 5c that PW triggering of the droplets did help form the lesions in the phantom with an input voltage of only 20 V, which failed when no PW was used. But the results also show that using PW to pre-trigger droplets does not always result in a larger lesion. For this droplet concentration and across the range of CW voltages used, the volumes of the thermal lesions produced by both treatments had no statistical difference (*p *> 0.05). The second image in Fig. 3a row 2 illustrates the bubble cloud shape created after PW alone for the 3.41 × 10^5^/mL droplet concentration. Comparing this to all the bubble clouds formed after combined treatment with the same PW but different CW voltages (Fig. 3b row 2), significant difference was found in echogenicity (*p* < 0.05), indicating the higher void fraction inside the bubble clouds formed after CW, which may have actually limited the size of the thermal lesions.

In summary, comparing to the CW only cases, the PW combined CW strategy could lower the CW input voltage required to create thermal lesions of similar volume, which could improve the safety of HIFU treatment. This would reduce the thermal dose to the overlying tissue and thus shorten cooling time required, leading to faster treatments. The PW combined CW strategy also led to larger volume of thermal lesion in the cases of the high droplet concentration. At the droplet concentration of 3.41 × 10^6^/mL, Fig. 5d shows that for the same CW exposure (10 s at 30 V), the volume of lesion produced by pre-triggering the droplets was 10 times larger, meanwhile no thermal lesion can be created in a phantom without droplets (Fig. 5a).

The results shown here have demonstrated that both the shape and the size of the thermal lesions can be changed by using the high amplitude short pulses of PW ultrasound before the significant thermal energy supplied by the CW ultrasound of HIFU. In real clinical treatments, the shape of the tumors are usually irregular. So to fully cover the tumor while sparing the important organs and normal tissues, a careful planning of the successive thermal lesions is critical since the lesion shapes are sensitive to the bubble clouds formed in the ADV technique. The PW strategy could help enlarge the thermal lesions or change the lesion shapes, which depends on the local droplet concentrations.

In this study, we used 200 pulses of PW ultrasound at an input voltage of 150 V to create a bubble area as large as possible. However, in real situations, both the number of pulses and the input voltage of the pulse could be adjusted to further manipulate the shape of the thermal lesions. Numerical models that couple the propagation of the acoustic waves, the dynamic formation of the bubble clouds and the thermal deposition and heat transfer would be a powerful treatment planning tool and aid in new treatment modality design. A numerical model has been proposed to calculate the ADV bubble cloud evolution during the PW exposure [25]. Simulation of the thermal lesion formation during the CW exposure in ADV assisted HIFU is now under investigation. This simulation work could be used for treatment planning in the future.

In this experiment, tissue-mimicking phantoms that can visualize thermal lesions were used to denote the thermal dose accumulated during the treatment, which is commonly used in experimental research on HIFU therapy [6, 11, 26]. The limitation of this approach is that it provides little detail about temperature distribution and variation during the treatment. In comparison, MRI can monitor the temperature distribution and record the temperature variation over time during the treatment, which is really helpful in understanding the complicated process of the highly dynamic interaction of the acoustic field, bubble cloud evolution and temperature field in ADV assisted HIFU treatment. In the future, a wider range of acoustic parameters would be needed to determine how robust the modeling performs under differing conditions. Therefore, more work needs to be done to reveal the underlying mechanism of this promising treatment technique and how to optimize this approach using ADV, which could in return help optimizing the treatment and benefit more patients.

## Conclusion

The influence of ADV bubbles on the formation of HIFU thermal lesions was investigated in this study. Two treatment strategies were examined, CW ultrasound heating alone and PW ultrasound combined with CW. It was found that with the same PW exposure, the bubble cloud created in phantom with high droplet concentration is much larger likely due to the strong reflection at the interface of high impedance difference. The shape of the bubble cloud progresses from small dots to teardrop to triangular with increasing concentrations of droplets. The cloud reproducibility was better at higher droplet concentrations.

The thermal lesions created in the phantoms with high droplet concentration were much larger in the case of PW combined with CW ultrasound treatment than those created by CW alone. By using the PW ultrasound to trigger the bubbles first, the thermal lesion created in the experiments varies from small dot to teardrop to vertically-oriented ellipsoid. It has been demonstrated that the size and morphology of the thermal lesion could be regulated by changes in both the acoustic pressure and the droplet concentration. The studies confirmed the feasibility of thermal lesion shape manipulation and offered new possibilities for optimization of HIFU treatment according to different requirements.

## Additional file

**Additional file 1.** Droplet size distribution. Droplet size distribution of the emulsion was measured by a Coulter counter.

## Abbreviations

ADV: acoustic droplet vaporization
HIFU: high intensity focused ultrasound
PW: pulse wave
CW: continuous wave
UCA: ultrasound contrast agent
