# Supplementary figures and images for: The effects on thermal lesion shape and size from bubble clouds produced by acoustic droplet vaporization
Source: Biomed Eng Online. 2018 Oct 29;17:163. doi: 10.1186/s12938-018-0596-z (PMC6206628; doi:10.1186/s12938-018-0596-z)

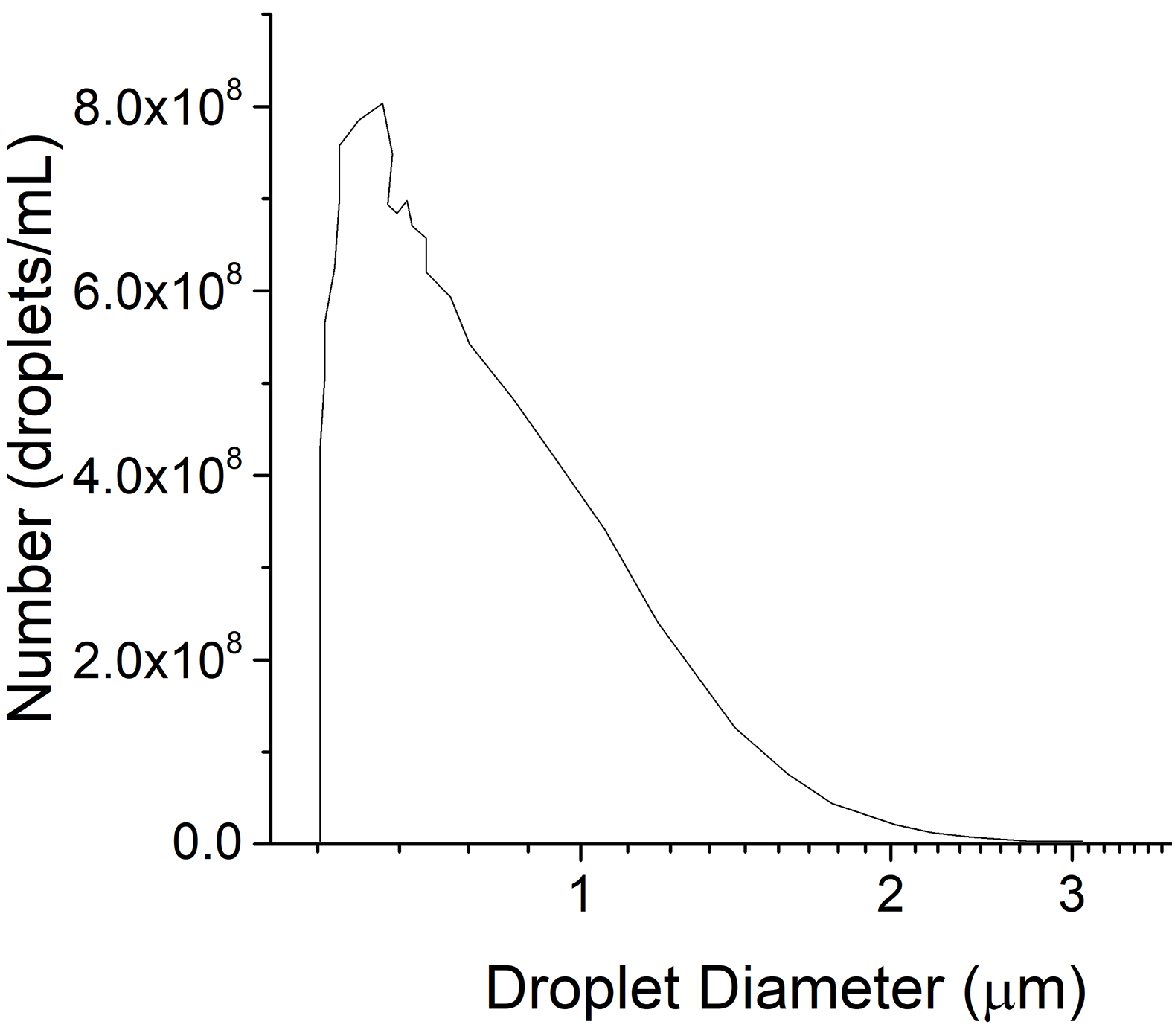

Supplement: Supplementary file 1 — Additional file 1. Droplet size distribution. Droplet size distribution of the emulsion was measured by a Coulter counter. [file 12938_2018_596_MOESM1_ESM.tif]
